# An optimized TOPS+ comparison method for enhanced TOPS models

**DOI:** 10.1186/1471-2105-11-138

**Published:** 2010-03-17

**Authors:** Mallika Veeramalai, David Gilbert, Gabriel Valiente

**Affiliations:** 1 Joint Center for Molecular Modeling, Sanford-Burnham Medical Research Institute, La Jolla, CA 92037, USA; 2 School of Information Systems, Computing and Mathematics, Brunel University, Uxbridge, Middlesex UB8 3PH, UK; 3 Algorithms, Bioinformatics, Complexity and Formal Methods Research Group, Technical University of Catalonia, E-08034 Barcelona, Spain

## Abstract

**Background:**

Although methods based on highly abstract descriptions of protein structures, such as VAST and TOPS, can perform very fast protein structure comparison, the results can lack a high degree of biological significance. Previously we have discussed the basic mechanisms of our novel method for structure comparison based on our TOPS+ model (Topological descriptions of Protein Structures Enhanced with Ligand Information). In this paper we show how these results can be significantly improved using parameter optimization, and we call the resulting optimised TOPS+ method as advanced TOPS+ comparison method i.e. *advTOPS+*.

**Results:**

We have developed a TOPS+ string model as an improvement to the TOPS [1-3] graph model by considering loops as secondary structure elements (SSEs) in addition to helices and strands, representing ligands as first class objects, and describing interactions between SSEs, and SSEs and ligands, by incoming and outgoing arcs, annotating SSEs with the interaction direction and type. Benchmarking results of an all-against-all pairwise comparison using a large dataset of 2,620 non-redundant structures from the PDB40 dataset [4] demonstrate the biological significance, in terms of SCOP classification at the superfamily level, of our TOPS+ comparison method.

**Conclusions:**

Our advanced TOPS+ comparison shows better performance on the PDB40 dataset [4] compared to our basic TOPS+ method, giving 90% accuracy for SCOP *alpha+beta*; a 6% increase in accuracy compared to the TOPS and basic TOPS+ methods. It also outperforms the TOPS, basic TOPS+ and SSAP comparison methods on the Chew-Kedem dataset [5], achieving 98% accuracy.

**Software Availability:**

The TOPS+ comparison server is available at http://balabio.dcs.gla.ac.uk/mallika/WebTOPS/.

## Background

The structural genomics consortium [6] aims to populate protein fold space using high-throughput experimental technologies. As a result the number of known structures in the Protein Data Bank (PDB) [7] is increasing rapidly every year and currently holds 59,790 structures (August 25, 2009). This highlights the importance of the need for fast and reliable protein structure comparison methods. There are various methods which use detailed 3D structures for comparison; SSAP [8,9] uses a double dynamic programming method that takes into account several different features of protein structure including phi/psi angles, accessibility and inter-residue vectors to align two protein structures. Other approaches include STAMP [10], DALI [11] and the Combinatorial Extension method [12]. On the other hand abstract level structural comparison methods are based on topological/vector models of secondary structure elements (SSEs) and their relationships. VAST is a vector based protein structure comparison method [13,14]. GRATH [15] is a graph-based algorithm that compares the axial vectors of alpha helices and beta strands of two proteins, together with the distances, angles and chirality between these vectors. It is based on a method by Grindley et al. [16]. Earlier work by Koch et al. [17] uses a graph method to find maximal common SSEs in a pair of proteins. TOPS is a graph-based method applied to the topological representation of the protein structures [3]. Although these methods perform very fast protein structure comparison in most cases the results have significantly less biological interpretation due to the abstract nature of the protein model. Moreover, the functional annotation problem is made much more complex by the fact that the number of protein folds is limited while their range of functions is very diverse. For example, the current version of the SCOP database classified the (single) TIM barrels protein fold into 33 distinct functional superfamilies.

This motivated our research to develop a novel topological model for protein structures, enhanced with structural and biochemical features, such as ligand interaction information and amino acid sequence length of the secondary structures, in order to permit better, more biologically significant comparison methods. Previously, we have discussed the basic mechanisms of our novel TOPS+ comparison method for novel topological models. We compute the edit distance between two proteins based on TOPS+ strings elements using a dynamic programming approach. We have benchmarked our method with an all-against-all pairwise comparison using a large dataset of 2,620 non-redundant structures from the PDB40 and the results were validated using the standard SCOP superfamily classification numbers. We have also compared our method against other methods and showed that it is faster than SSAP, FATCAT, DALI and TOPS and that it has a comparable performance against TOPS [18]. Recently we developed the TOPS++FATCAT system that exploits the TOPS+ strings comparison method to speedup the FATCAT protein structural alignment program for fast flexible structural alignment, while preserving the accuracy of the original FATCAT method [19]. These promising results have facilitated the introduction of further constraints on ligand-arc matching.

In this paper, we show how the above results can be significantly improved using parameter optimization at two stages of the TOPS+ method: (i) in the generation of the dynamic programming table and (ii) in the computation of the comparison score using a compression measure. The dynamic programming algorithm includes weight tables for matching TOPS+ strings elements, the match scores take into account not only the SSEType, orientation but in addition they include scores for total in/out/ligand arcs together with their arc types such as right and left chiralities, and parallel and anti-parallel hydrogen-bonds. This research work involved (a) generating the TOPS descriptions enhanced with in/out/ligand arc information for a large set of proteins; (b) designing the weight tables; (c) optimization of weights in the table; (d) designing a pairwise comparison metric based on a compression measure and optimizing different parameters to take into account the variability on both components of the topological and ligand interaction features. The optimization of our advanced TOPS+ comparison method was carried out on the PDB40 representative dataset. Furthermore, we assess the biological significance of our method against existing protein structure comparison methods based on cluster analysis and validation using an *F*-measure calculation [20,21] on the Chew-Kedem dataset [4,5].

## Results and Discussion

### Analysis of results for the PDB40 dataset

**Figure 1:**
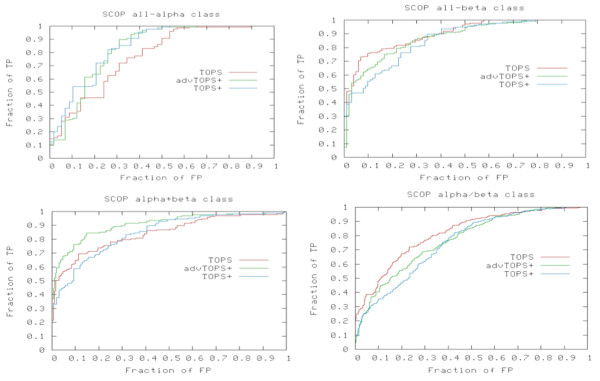
**ROC curves for the PDB40 dataset**. ROC curves for SCOP classes *all-alpha*, *all-beta*, *alpha/beta *and *alpha+beta *from TOPS, TOPS+ and advanced TOPS+ methods on the PDB40 dataset.

Figure 1 shows the ROC curves and Table 1 gives AUC values for SCOP classes *all-alpha*, *all-beta*, *alpha/beta *and *alpha+beta *on the PDB40 subset dataset obtained from the TOPS, TOPS+ and advanced TOPS+ (advTOPS+) methods. The results show that the advTOPS+ method is superior to TOPS+ on classes *all-beta*, *alpha/beta *and *alpha+beta *with 82%, 77% and 90% accuracy (see Table 1); while it gives similar results on the *all-alpha *class with an accuracy of 82%. When we compared our advTOPS+ method with TOPS we have better performance on *alpha+beta *and *all-alpha *classes with accuracy level increased by 6%; the result is comparable in the case of classes *all-beta *and *alpha/beta *protein domains. The TOPS method relies on arc information and in alpha rich proteins there are no hydrogen-bond arcs and few chirality arcs, hence it performs poorly on the *all-alpha *class. Our TOPS+ methods has a better performance compared to TOPS in *all-alpha *class of proteins, because in our TOPS+ model we have included additional biochemical features such as loops, SSE-ligand interactions, and SSE segment length. Moreover in the *all-alpha *class, most of the proteins have structure-dependent ligand interactions such as DNA-binding proteins with (Helix-Loop-Helix = HLH, Helix-Turn-Helix = HTH) and metal-binding proteins (like HEM--binding proteins, etc.). Thus our method can recognize those proteins more efficiently compared to TOPS.

**Table 1:** ROC curve and F-measure analysis of structural homology for the PDB40 dataset.

SCOP Class		TOPS	TOPS+	advTOPS+
1	All alpha	0.76/0.79	0.83/0.85	0.82/0.88
2	All beta	0.89/0.85	0.85/0.83	0.87/0.86
3	Alpha/beta	0.82/0.75	0.75/0.70	0.77/0.70
4	Alpha+beta	0.84/0.75	0.84/0.74	0.90/0.81

AUC/F-measure values for all the comparison methods for the PDB40 dataset.

In *alpha+beta *class our advTOPS+ method has a 90% accuracy, which is superior when compared with both TOPS and our basic TOPS+ method, which have only 84% accuracy (see Table 1). Because these proteins are composed of segregated alpha and beta regions the structure-dependent ligand interactions and additional chiral, hydrogen bonds are also present. Thus our parameter optimization can handle all arcs more efficiently.

On the other hand, the *alpha/beta *class of proteins contains mixed alpha and beta secondary structures; more importantly although the protein domains from these classes have ligand interactions, they *may not be structure-dependent ligand interactions*. In these classes for most of the protein superfamilies the ligands have a tendency to bind the clefts or binding pocket which have appropriate *physiochemical *properties and the correct conformational geometry of the amino acids. Furthermore it is important to note that in our TOPS+ and advTOPS+ comparison methods we have considered only the total number of ligand-arcs rather than the actual ligand property match, thus we have false positives in some SCOP classes. In the case of all-beta class proteins our advTOPS+ method has comparable performance against TOPS with 87% accuracy (see Table 1); in this class proteins contain a significant number of hydrogen bond and chiral arcs, and thus parameter optimization is performed more efficiently. From the F-measure statistical evaluation analysis (we used the same cutoff value of 0.35 for all three methods) we found that the advTOPS+ method appears to always do better than TOPS and TOPS+ except for the *alpha/beta *class of proteins (see Table 1).

The overall results show that our advTOPS+ method exhibits substantial improvement compared to basic TOPS+. It has better performance for *all-alpha *and *alpha+beta *proteins compared to TOPS. On the other SCOP classes the performance is comparable with TOPS. Since our method considers only the total number of ligand arcs rather than the actual ligand property this leads to false positives to some extent. Our advTOPS+ method can efficiently recognize structure-dependent ligand interactions appropriately in the case of DNA-binding proteins and metal binding proteins.

### Analysis of results for the Chew-Kedem dataset

**Figure 2:**
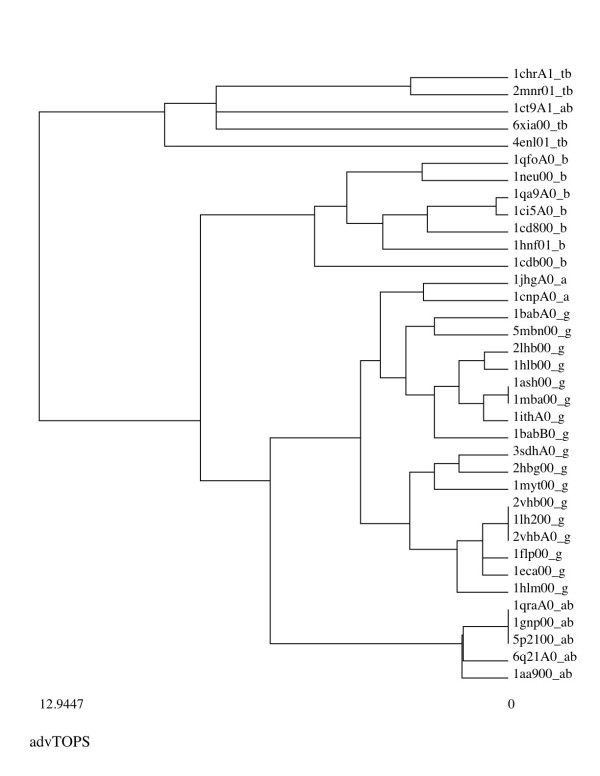
**Clusters for the Chew-Kedem dataset**. Chew-Kedem dataset clusters obtained from the advanced TOPS+ comparison method (for more information see the supplementary material page at http://balabio.dcs.gla.ac.uk/mallika/WebTOPS/optTOPSplus-results.html).

Our advanced TOPS+ comparison method outperforms all the other methods (TOPS, basic TOPS+ and SSAP) and groups 36 representative proteins from five fold families into biologically significant clusters (see Figure 2). In Figure 2 each protein domain is represented in the following format "domainName_foldFamilies". The tags of the "foldFamilies" represents protein structures from five different protein folds; where tb = TIM barrel, g = Globins, ab = alpha beta, b = all beta, and a = all alpha protein families. The clusters obtained from our advanced TOPS+ method show that all of the protein domains are grouped according to their structural fold and biological significance except *d1ct9a1 *(see explanations below). The basic TOPS+ method also groups most of the protein domains into correct fold families except for an all beta protein *d1cdb__ *and two protein domains *d2hbg__, d1hlm__ *from globins which are grouped together with all alpha proteins (see the supplementary material page for clusters from other methods and additional information at http://balabio.dcs.gla.ac.uk/mallika/WebTOPS/optTOPSplus-results.html). In comparison both the TOPS and SSAP methods produce clusters in which there are many wrongly grouped domains (see supplementary material page for clusters from other methods and additional information at http://balabio.dcs.gla.ac.uk/mallika/WebTOPS/optTOPSplus-results.html). The quantitative analysis using the *F*-measure calculation results in more than 98% accuracy for our advTOPS+ method, which is higher than SSAP (96%), TOPS (95%) and basic TOPS+ (93%) (see Table 2). When we compare our advTOPS+ with our basic TOPS+ method we have achieved a 5% improvement based on parameter optimization.

**Table 2:** Biological significance of protein domain clusters for the Chew-Kedem dataset.

Method	*F*-measure
SSAP	0.966
TOPS	0.955
TOPS+	0.931
advTOPS+	0.985

*F*-measure of the clusters obtained from different protein structure comparison methods.

**Figure 3:**
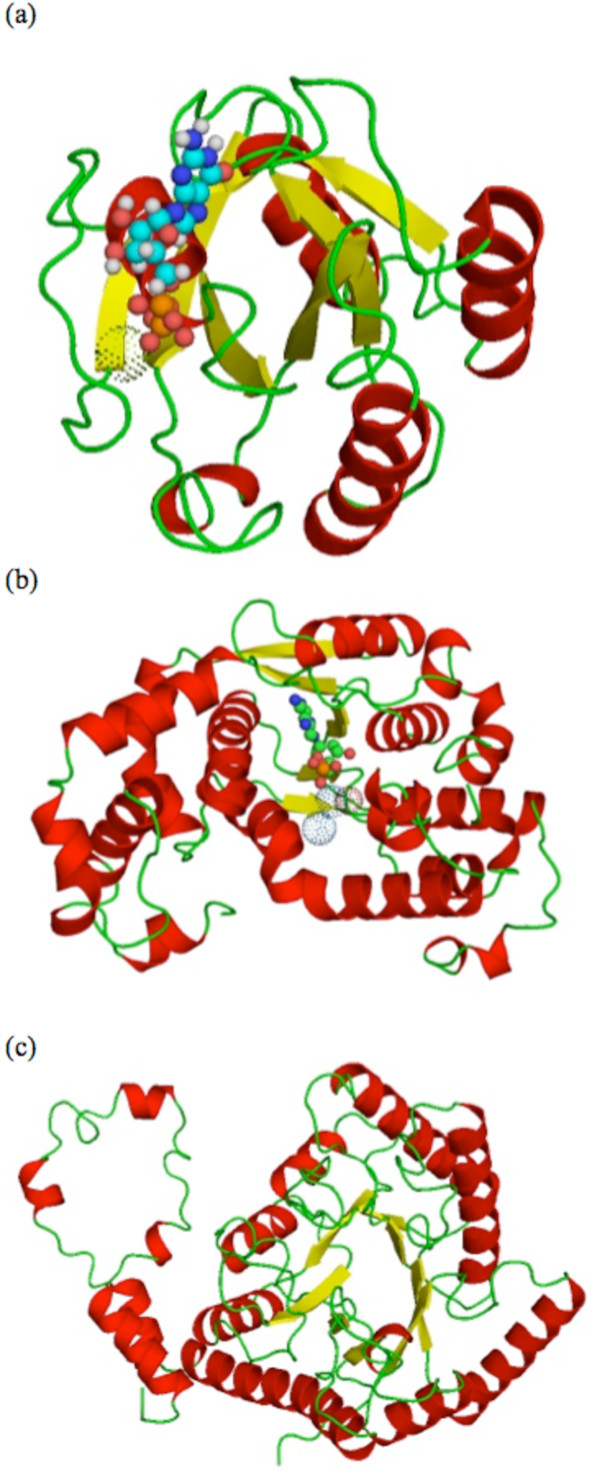
**Protein domain 3D cartoon diagrams**. 3D cartoon diagrams (top view) of the alpha-beta protein domains (a) *d1aa9__*, (b) *d1ct9a1*, (c) TIM barrel protein domain *d6xia__*. Beta-strands, alpha-helices and loops are colored with yellow, red and green, respectively. Ligand molecules are indicated by spheres and dots (metals).

One interesting example from our cluster analysis (see Figure 2) is that of the Asparagine synthetase B, C-terminal domain *d1ct9a1 *(alpha-beta fold) which is grouped by the advanced TOPS+ method with the TIM barrel domains (see Figure 3). Table 3 provides advanced TOPS+ comparison scores for *d1ct9a1 *(SSE_Ln = 53) against those protein domains belonging to alpha-beta and TIM-barrel fold families, together with their SSEs length (SSE_Ln) and LCS pattern length (LCS PAT_Ln). Interestingly, *d1ct9a1 *has a smaller distance score against all of the TIM-barrel proteins compared to alpha-beta protein domains; specifically, for the xylose isomerase protein *d6xia__*. When we closely checked the 3D structure of *d1ct9a1*, we found that it has alpha helices in its N-terminal domain and it contains half TIM-barrel like structures in its C-terminal domain (see Figure 3). This suggests that a sub-domain of the *d1ct9a1 *exhibits a structural drift between the alpha-beta fold and the TIM-barrel fold. The structural drift [22] is a special case of gregariousness as described by [23]. Since our TOPS+ method is based on an abstraction of protein structure in the form of SSEs and topological features without any geometrical properties it is able to perform matching at the fold level and to include most of the SSEs which are common to two protein structures.

**Table 3:** advTOPS+ comparison scores for the Chew-Kedem dataset.

Protein Fold	Domain	SSE Ln	LCS PAT Ln	Adv TOPS+ Score	LCS SSE PATTERN
Alpha-beta	d1aa9__	23	20	0.49	uEUhuEuhuEuhuEUhuEhu
	d1gnp__	25	20	0.51	uEUhuEhuEuhuuEUhuEhu
	d6q21a_	21	16	0.59	uEUhuEuhuEUhuEhu
	d1qraa_	25	20	0.51	ueUHueHueuHuueUHueHu
	d5p21__	25	20	0.51	ueUHueHueuHuueUHueHu

TIM-barrel	d6xia__	60	40	0.30	uhuhuHuHueuHueuHuhuehhuHuHueuuuHuhuhuuhu
	d2mnr_1	37	29	0.37	uuuuHueuHueuHueuHueuHuuhuuuhu
	d1chra1	41	31	0.35	uuuHuHueuHueuHueuHuHueuHuhuuuhu
	d4enl_1	55	35	0.37	uhuhuHuueuuuHuhueuHuHuuhuuHuuHuHuhu

advTOPS+ comparison scores for alpha-beta and TIM-barrel folds in the Chew-Kedem dataset.

## Conclusions

In this paper we have reported the generation of TOPS+ and TOPS+ strings models for large datasets and have presented an improved TOPS+ comparison method using parameter optimization both for the computation of the dynamic programming table and the computation of the comparison score using a compression metric. Through our evaluation analysis we have showed that our advanced TOPS+ comparison method has a substantial improvement on all the SCOP classes compared to our basic TOPS+ method. Our advanced TOPS+ method has better performance compared to TOPS on *alpha+beta *and *all-alpha *and is comparable on *all-beta *and *alpha/beta*. On the Chew-Kedem dataset our advanced TOPS+ comparison outperforms all the other methods.

This demonstrates that our TOPS+ and TOPS+ strings models can find more biologically significant results and has led to interesting new directions to incorporate ligand-pattern discovery in TOPS+ comparison [24]. Our method is faster than TOPS and SSAP because it has time complexity *O*(*n*^2^), where *n *represents the number of SSEs in the protein domains. This research opens new doors to an exciting improvement to our TOPS+ models and advanced TOPS+ comparison method by the addition of features such as amino-acid sequences, biochemical properties of the protein-ligand interaction at atomic level, and arc scores (both topological level and ligand level) for each SSE. Moreover we can improve the comparison process with additional statistical scoring values for each TOPS+ strings element match both at the micro (atomic-details of protein-ligand interaction information) and the macro level (abstract level).

Furthermore our novel TOPS+ models, TOPS+ strings and comparison approaches could be applicable to different problem areas such as RNA secondary structure comparison and prediction. Most of the drug-discovery process starts with in-silico chemical compound screening which is computationally expensive. Our TOPS+ comparison approach could be applied as an initial step to prune the search space and filter the proteins into same folds interacting with similar or different ligands and different folds interacting with similar or different ligands.

## Methods

### TOPS+ and TOPS+ Strings Models

The TOPS model [1,2,25,26] represented protein structures at the fold level by a graph where the nodes stand for SSEs--(up or down) alpha-helices and beta-strands--and (non-directed) edges represent right or left-handed chirality and parallel or anti-parallel hydrogen-bond relationships. In addition, there is a total ordering over the nodes, corresponding to the backbone of the protein. Our TOPS+ model enhances the original TOPS graph model with structural and biochemical features such as ligand interaction information and amino acid sequence length of the secondary structures. We have added extra nodes for loops (represented as a first class object--SSE) and ligands as well as maintaining the existing nodes for alpha-helices and beta-strands.

Further, we have designed a string model based on our TOPS+ graph model where the long-range and short-range interactions between the SSEs are converted into incoming and outgoing arcs for each SSE, which maintain the directions and arc type properties. All relevant SSE nodes are enhanced with SSE-ligand interaction information which includes loop-ligand interaction information. We abstract away from the ligands themselves, to give a linear model called TOPS+ string which preserves the essential biochemical information whilst permitting more efficient and non-heuristic algorithms for comparison.

**Figure 4:**
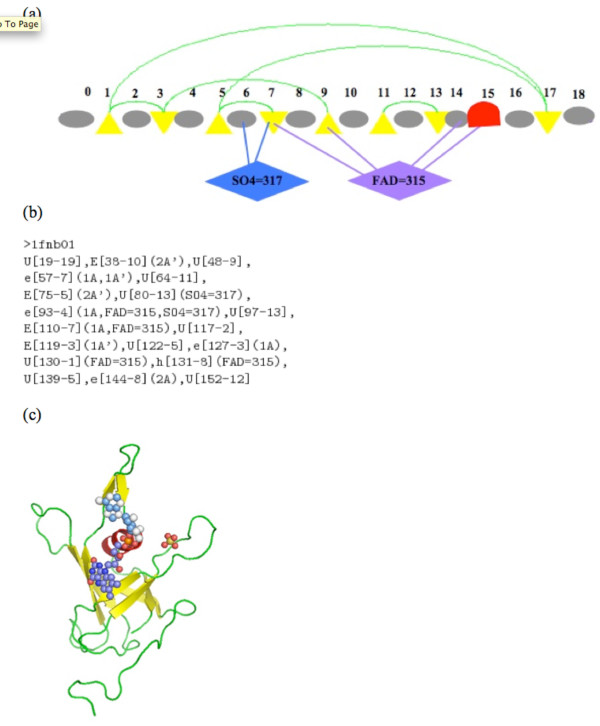
**TOPS+ representation of a protein domain**. TOPS+ diagram and string representation of the protein domain *1fnb01*. (a) TOPS+ diagram. (b) TOPS+ string. (c) 3D cartoon.

In detail, each SSE node is enriched with SSEType, SSE segment details are indicated by PS-SL where PS is the PDB start number and SL is the SSE segment length, total incoming arcs (InArc) and total outgoing arcs (OutArc), total number of ArcTypes, and total number of ligand arcs (LigArc). The SSEType is given by {E, e, H, h, U, u} where, 'E' and 'e' represent the 'up' and 'down' oriented beta-strands; 'H' and 'h' indicate the 'up' and 'down' oriented alpha-helices; 'U' and 'u' represent ligand-bound and ligand-free loops. The InArcType is represented as a {R, L, P, A}, where 'R' and 'L' represent right and left chiralities; 'P' and 'A' represent parallel and anti-parallel hydrogen-bonds respectively. The OutArcType is represented in a similar manner by {R', L', P', A'}. Ligand arcs are indicated by LT = AA where LT is the ligand type and AA is the PDB number. For example, Figure 4(a) and 4(b) shows the representation of the TOPS+ model and the abstract TOPS+ string representation for the protein domain *1fnb01 *respectively. Here the triangles represent the beta strands; red curves represent the alpha helix; circles indicate loop regions and green arcs indicate hydrogen bonds between two beta strands, called the anti-parallel beta sheet. In the 3D cartoon of *1fnb01 *shown in Figure 4(c), the ligand molecules 'FAD' and 'SO4' are indicated by spheres. We have computed the topological information for a given PDB based on either SCOP or CATH domain definitions by using the TOPS cartoon generation method [27]. The InterCal program (Wallace and Michalopoulos, personal communication) provides the protein-ligand interaction information. Thus, by combining topological details and the protein-ligand interaction information, we have constructed our TOPS+ and TOPS+ strings models for a given protein domain; for more details refer to Veeramalai and Gilbert [18]. We have generated the TOPS+ model and the TOPS+ strings representation of 28 976 and 28 298 protein domains including 16 163 and 14 887 ligand-bound protein domains corresponding to the CATH 2.4 and the SCOP 1.61, respectively.

### Advanced TOPS+ Comparison Method based on Dynamic Programming Algorithm

Our TOPS+ comparison method computes a comparison score between two proteins based on edit distance using a dynamic programming approach. The *Levenshtein distance *or *edit distance *[28,29] gives a measure (the cost) of the minimum number of elementary edit operations (insertions, deletions and substitutions of characters) necessary to transform one sequence into the other. In this research we have improved our existing method using parameter optimization in the dynamic programming table computation and also in the computation of the comparison score.

We have added some additional functions to the standard edit distance algorithm in order to compare the TOPS+ strings models of the two protein structures. Our optimized comparison method comprises five major steps as follows, where steps (1) and (5) take parameter tuning tables and penalty weights for arc information (both topological arcs and ligand arcs) into account:

1. Recursive definition of the optimal dissimilarity score for match and mismatch between TOPS+ strings elements (this process is based on the advanced_SSEArc+Match function, which incorporates the parameter optimization process using parameter tuning table).

2. Construction of the Edit Distance (ED) matrix (dynamic programming table).

3. Trace-back on the ED matrix (dynamic programming table).

4. Obtain the LCS (Longest Common Substring), which is equivalent to the largest common structural core.

5. Computation of the comparison score based on the compression measure which is optimized with penalty weights for arc information (at both topological arcs and ligand arcs).

In our optimized TOPS+ comparison method, the computation of the edit distance matrix *M *is an important process, in which the advanced_SSEArc+Match function plays a key role in assigning dissimilarity scores for each TOPS+ strings element match or mismatch between the target *t*_*i *_∈ *T *and the source *s*_*j *_∈ *S*. This function handles the parameter optimization process while computing the construction of the edit-distance matrix using a dynamic programming approach. It takes the basic parameter list *P*_*b *_supplied together with the input and constructs the parameter tuning table *PT *with 12 weights (*w*_1 _to *w*_12_) and integrates these weights with the absolute arc differences (*D*_1 _to *D*_12_) between the TOPS+ strings elements *t*_*i *_∈ *T *and *s*_*j *_∈ *S*, computing the final normalized dissimilarity score for match or mismatch between the *t*_*i *_∈ *T *and *s*_*j *_∈ *S*. In each step the advanced_SSEArc+Match function performs the following processes in order to obtain the dissimilarity scores between each pair of TOPS+ strings elements of *T *and *S *and to construct the dynamic programming table:

• Construct the parameter tuning table *PT *based on the basic parameter list *P*_*b *_and this process performed once.

• Compute the absolute difference for the arc features such in/out/ligand arc between *t*_*i *_and *s*_*j *_of *T *and *S *respectively.

• Compute the optimized dissimilarity score for *t*_*i *_and *s*_*j *_match using equations (1) and (2) below.

• Construct the dynamic programming table.

**Algorithm 1 (Edit distance between TOPS+ strings) ***A function call ComputeEditDistance*(*T, S*) *computes the edit distance matrix M, the backtrace pointer matrix P, the edit distance value ed, and the longest common subsequence lcs of two TOPS+ strings T and S*.

   **function ***ComputeEditDistance*(*T *= *t*_1_, ..., *t*_*n*_, *S *= *s*_1_, ..., *s*_*m*_)

      *M *[0, 0] ← 0

      **for ***i *← 1, ..., *n ***do**

         *M *[*i*, 0] ← *i*

      **for ***j *← 1, ..., *m ***do**

         *M *[0, *j*] ← *j*

      **for ***i *← 1, ... *n ***do**

         **for ***j *← 1, ..., *m ***do**

            *A *← *SSEArc+Match*(*t*_*i*_, *s*_*j*_)

            *M *[*i*, *j*] ← min{*M *[*i*, *j *- 1] + 1, *M *[*i*, *j *- 1] + 1, *M *[*i *- 1, *j *- 1] + *A*}

            **if ***M *[*i*, *j*] = *M *[*i *- 1, *j *- 1] + *A ***then**

               *P *[*i, j*] ← '*m*'   ▷ match or mismatch of *s*_*i *_to *t*_*j*_

            **else if ***M *[*i*, *j*] = *M *[*i*, *j *- 1] + 1 **then**

               *P *[*i*, *j*] ← '*i*'   ▷ insertion of *s*_*j *_into *t*

            **else**

               *P *[*i*, *j*] ← '*d*'   ▷ deletion of *t*_*i *_from *t*

      *ed *← *M *[*n, m*]

      *lcs *← *BuildLCS*(*M, P, T, S*)

      **return **⟨*M, P, ed, lcs*⟩

   **function ***BuildLCS*(*M, P, T, S*)

      *lcs *← ∅   ▷ empty sequence

      *k *← 0

      *i *← *n *▷ length of *T*

      *j *← *m *▷ length of *S*

      **while ***i *> 0 **or ***j *> 0 **do**

         **if ***P *[*i*, *j*] = '*m*' **then**

            *lcs *← *lcs *∪ *t*_*j*-1 _▷ match or mismatch of *s*_*i*-1 _to *t*_*j*-1_

            *k *← *k *+ 1

            *i *← *i *- 1

            *j *← *j *- 1

         **else if ***P *[*i*, *j*] = '*d*' **then**

            *i *← *i *- 1   ▷ deletion of *t*_*i*-1 _from *t*

         **else**

            *j *← *j *- 1   ▷ insertion of *s*_*j*-1 _into *t*

      **return ***lcs*

   **function ***SSEArc+Match*(*t*_*i*_, *s*_*j*_)

      *mS *← 0

      *Parse*(*t*_*i*_, *t*_*sk*_, *t*_*I*_, *t*_*O*_, *t*_*L*_, *t*_*IR*_, *t*_*IL*_, *t*_*IP*_, *t*_*IA*_, *t*_*OR*_, *t*_*OL*_, *t*_*OP*_, *t*_*OA*_)

      *Parse*(*s*_*j*_, *s*_*sk*_, *s*_*I*_, *s*_*O*_, *s*_*L*_, *s*_*IR*_, *s*_*IL*_, *s*_*IP*_, *s*_*IA*_, *s*_*OR*_, *s*_*OL*_, *s*_*OP*_, *s*_*OA*_)

      **if ***MatchSSEArc+features*(*t*_*i*_, *s*_*j*_) **then**

         *mS *← *mS *+ 1

      **return ***mS*

   **procedure ***Parse*(*t*_*i*_, *t*_*sk*_, *t*_*I*_, *t*_*O*_, *t*_*L*_, *t*_*IR*_, *t*_*IL*_, *t*_*IP*_, *t*_*IA*_, *t*_*OR*_, *t*_*OL*_, *t*_*OP*_, *t*_*OA*_)

      *t*_*sk *_← *secondary structure length of t*_*i*_

      *t*_*I*_, *t*_*O*_, *t*_*L *_← *total number of incoming, outgoing, ligand arcs of t*_*i*_

      *t*_*IR*_, *t*_*IL*_, *t*_*IP*_, *t*_*IA *_← *total number of incoming arcs of type R, L, P, A of t*_*i*_

      *t*_*OR*_, *t*_*OL*_, *t*_*OP*_, *t*_*OA *_← *total number of outgoing arcs of type R, L, P, A of t*_*i*_

The time complexity is *O*(*n*^2^) where *n *is the length of the string of SSEs. The current version of our TOPS+ method performs global alignment [30]. However, local alignment [31] can be applied to find the local structural similarity or patterns such as similar SSE-ligand interactions at local level across different folds.

### Optimizing the Computation of the Dynamic Programming Table

We performed parameter tuning/optimization in order to obtain the optimal approximate match between two protein structures. In general, at the superfamily level, only core structures are conserved throughout evolution across the members of protein families. Studies have shown that the number of SSE insertions and deletions is variable for different sequence families or organisms [32]. This implies that variable numbers of 'indels' are applicable to the ArcsTypes and SSETypes across protein families from various organisms within a superfamily. Thus, it is important to develop a cost matrix with an additional penalty scoring function for such an approximate matching process. In the following sections we discuss the development of the parameter tuning table and the computation of the absolute difference between ArcTypes types and SSETypes. Subsequently, we explain the main parameter optimization process involved in the computation of the dynamic programming table, which exploits the computation of a normalized dissimilarity score for TOPS+ strings element match.

#### Development of Parameter Tuning Table

The parameter-tuning table *PT *contains a list of weights for all the SSEArc+ features given by *w*_1_, ..., *w*_12_, where, *w*_1 _is applied to SSEType; *w*_2 _to total incoming arcs; *w*_3_, *w*_4_, *w*_5 _and *w*_6 _to total incoming arcs of type R, L, P and A respectively; *w*_7 _to total outgoing arcs; *w*_8_, *w*_9_, *w*_10 _and *w*_11 _to total outgoing arcs of type R, L, P and A respectively; and *w*_12 _to total ligands arcs. Every element 'y' in *PT *is in the range 1..9. The SSEType weight *w*_1 _is obtained from the ISM or DSM scoring matrices (see Table 4). All the other weights *w*_2 _to *w*_12 _are computed using a basic parameter set *P*_*b *_which is supplied with the input. The basic parameter *P*_*b *_consists of a list of five positive integers in the range 1..9 given by *P*_*b *_= (*p, q, r, s, t*). The parameter values *p *and *q *correspond to the parallel and the anti-parallel hydrogen-bond arcs respectively; *r *and *s *correspond to right-handed and left-handed chiralities respectively; *t *is for the ligand arcs. Based on these conditions, we have constructed a total of 1,134 unique lists of basic parameters *P*_*b *_used for tuning the parameter table *PT *with weights as follows:

**Table 4:** Identity and dissimilarity scoring matrices for TOPS+ diagrams.

	ISM						DSM					
**SSE**	**E**	**e**	**H**	**h**	**U**	**u**	**E**	**e**	**H**	**h**	**U**	**u**

E	0	1	1	1	1	1	0	1	2	2	2	2
e	1	0	1	1	1	1	1	0	2	2	2	2
H	1	1	0	1	1	1	2	2	0	1	2	2
h	1	1	1	0	1	1	2	2	1	0	2	2
U	1	1	1	1	0	1	2	2	2	2	0	1
u	1	1	1	1	1	0	2	2	2	2	1	0

Identity Scoring Matrix (ISM) and Dissimilarity Scoring Matrix (DSM) of secondary structure elements used for matching TOPS+ diagrams.

• *w*_3 _= *w*_8 _= *r *(for incoming and outgoing arc type_R)

• *w*_4 _= *w*_9 _= *s *(for incoming and outgoing arc type_L)

• *w*_5 _= *w*_10 _= *p *(for incoming and outgoing arc type_P)

• *w*_6 _= *w*_11 _= *q *(for incoming and outgoing arc type_A)

• *w*_2 _= *w*_7 _= *r *+ *s *+ *p *+ *q *(for total incoming and outgoing arcs)

• *w*_12 _= *t *(for total ligand arcs)

#### Computation of Absolute Differences

The normalized dissimilarity score for SSE is obtained from the absolute difference of the SSE length (amino acid sequence length of the SSE) between the TOPS+ strings elements *t*_*i *_∈ *T *and *s*_*j *_∈ *S *given by,(1)

where, *t*_*sk *_and *s*_*sk *_are the secondary structure length for *t*_*i *_∈ *T *and *s*_*j *_∈ *S *respectively. The absolute differences of the total number of incoming, outgoing and ligand arcs between the TOPS+ strings elements *t*_*i *_∈ *T *and *s*_*j *_∈ *S *are computed based on the equations given in Table 5. This process includes the computation of the absolute arc differences for the incoming and outgoing arcs according to their ArcTypes {R, L, P, A} between *t*_*i *_∈ *T *and *s*_*j *_∈ *S *of the TOPS+ strings elements.

**Table 5:** Normalized similarity score between secondary structure elements.

Absolute Differences	Equations	Description
total incoming arcs	*D*_2 _= |*t*_*I *_- *s*_*I*_|	*t*_*I *_and *s*_*I *_are the total number of incoming arcs of the TOPS+ strings elements *t*_*i *_∈ *T *and *s*_*j *_∈ *S *respectively

total incoming arcs type_R	*D*_3 _= |*t*_*IR *_- *s*_*IR*_|	*t*_*IR *_and *s*_*IR *_indicate the total number of incoming arcs type_R for *t*_*i *_∈ *T *and *s*_*j *_∈ *S *respectively

total incoming arcs type_L	*D*_4 _= |*t*_*IL *_- *s*_*IL*_|	*t*_*IL *_and *s*_*IL *_indicate the total number of incoming arcs type_L for *t*_*i *_∈ *T *and *s*_*j *_∈ *S *respectively

total incoming arcs type_P	*D*_5 _= |*t*_*IP *_- *s*_*IP*_|	*t*_*IP *_and *s*_*IP *_indicate the total number of incoming arcs type_P for *t*_*i *_∈ *T *and *s*_*j *_∈ *S *respectively

total incoming arcs type_A	*D*_6 _= |*t*_*IA *_- *s*_*IA*_|	*t*_*IA *_and *s*_*IA *_indicate the total number of incoming arcs type_A for *t*_*i *_∈ *T *and *s*_*j *_∈ *S *respectively

total outgoing arcs	*D*_7 _= |*t*_*O *_- *s*_*O*_|	*t*_*O *_and *s*_*O *_are the total number of outgoing arcs for the TOPS+ strings elements *t*_*i *_∈ *T *and *s*_*j *_∈ *S *respectively

total outgoing arcs type_R	*D*_8 _= |*t*_*OR *_- *s*_*OR*_|	*t*_*OR *_and *s*_*OR *_indicate the total number of outgoing arcs type_R for *t*_*i *_∈ *T *and *s*_*j *_∈ *S *respectively

total outgoing arcs type_L	*D*_9 _= |*t*_*OL *_- *s*_*OL*_|	*t*_*OL *_and *s*_*OL *_indicate the total number of outgoing arcs type_L for *t*_*i *_∈ *T *and *s*_*j *_∈ *S *respectively

total outgoing arcs type_P	*D*_10 _= |*t*_*OP *_- *s*_*OP*_|	*t*_*OP *_and *s*_*OP *_indicate the total number of outgoing arcs type_P for *t*_*i *_∈ *T *and *s*_*j *_∈ *S *respectively

total outgoing arcs type_A	*D*_11 _= |*t*_*OA *_- *s*_*OA*_|	*t*_*OA *_and *s*_*OA *_indicate the total number of outgoing arcs type_A for *t*_*i *_∈ *T *and *s*_*j *_∈ *S *respectively

total ligand arcs	*D*_12 _= |*t*_*L *_- *s*_*L*_|	*t*_*L *_and *s*_*L *_are the total number of ligand arcs for the TOPS+ strings elements *t*_*i *_∈ *T *and *s*_*j *_∈ *S *respectively

Equations used for computing the normalized similarity score between secondary structure elements of TOPS+ strings.

The absolute differences for total incoming arcs are given by,(2)

where *t*_*I *_and *s*_*I *_are the total number of incoming arcs of the TOPS+ strings elements *t*_*i *_∈ *T *and *s*_*j *_∈ *S *respectively. The absolute arc differences between incoming arcs, outgoing arcs and their arc types are calculated based on Table 5.

#### Computation of Normalized Dissimilarity Score

We have combined the parameter tuning table *PT *and the absolute arc differences to compute the final normalized dissimilarity score *mSn *for the matching or mismatching of the TOPS+ strings elements *t*_*i *_∈ *T *and *s*_*j *_∈ *S*. The general formula for calculating the normalized dissimilarity score is given in equation (3), where all the weights *w*_*i *_are obtained from the parameter tuning table *PT*; all the values for absolute arc differences *D*_*i *_are computed based on equation (2) and Table 5. Since the SSETypes {H, h} do not have any Hbond arcs {P, A}, we have substituted zero weights for corresponding arc weights as follows: *w*_*IP *_= *w*_*IA *_= *w*_*OP *_= *w*_*OA *_= 0. Note that this condition is applicable only when both *t*_*i *_∈ *T *and *s*_*j *_∈ *S *have SSEType {H, h}. The final normalized dissimilarity score for the helix SSETypes helix_mSn is calculated using equation (5). A similar condition is also applicable to the loops; they do not have any other arcs except their ligand arcs and their normalized similarity score loop_mSn is given by equation (6). The normalized dissimilarity score strand_mSn for the beta strands is calculated using equation (4). For all the other non-match SSEType we have considered the weights obtained from the ISM or DSM scoring matrices (see Table 4). When we applied these dissimilarity scores to loops we consider both 'u' (loop without ligand interaction) and 'U' (loop with ligand interaction) as identical. However when we perform ligand pattern discovery in a different context (for instance, similar ligand interaction on different fold types) for each SSE we have considered them as different SSETypes [24].(3)

### Computation of the Optimized Comparison Score (metric)

We have computed a pairwise comparison score based on a compression measure (itself a metric) to evaluate the goodness of patterns for a set of TOPS+ strings models of the proteins. This procedure was adapted from Brazma et al. [33] and has been employed in the TOPS comparison and pattern discovery methods [1,2,26]. We have calculated the compression value based on the total number of proteins in the given set (in this case there are two proteins), the total number of SSEs in the proteins in the given input set and total number of SSEs in the common pattern. Similarly, we also take into account arc information such as total number of in/out/ligand arcs in the given set of proteins as well as the same information for the LCS pattern. The raw compression value over SSEs is given as:(7)

where, |*S*_*i*_| is the total number of SSEs in a protein *i*, *n *is the total number of proteins in the target set and |*S*_*p*_| is the total number of SSEs in the LCS pattern. The normalized compression score over SSEs, which varies from 1 (best) to 0 (worst), is computed by:(8)

Similarly we compute the normalized compression (nC) score for InArcs, OutArcs and LigandArcs. The overall normalized TOPS+ comparison score for TOPS+ strings models is computed by (9), combining the compression for the SSEs and arc type, optimized by different weights *k*_1_, ..., *k*_4 _(see below).(9)

We have computed 17 different combinations of compression values based on ED and LCS together with or without different levels of SSEArc+ features information. Supplementary Table 1 (see supplementary material page at http://balabio.dcs.gla.ac.uk/mallika/WebTOPS/optTOPSplus-results.html) gives all the 17 normalized compression scores we have calculated based on ED and LCS from our advanced TOPS+ comparison with (output order of the results) and description.

We have performed training and analysis of our advanced TOPS+ comparison method with the parameter tuning table. Our method incorporates parameter optimization at two levels, both in the computation of the dynamic programming table and in the computation of the normalized compression measure. We have tested our method with 1,134 unique basic parameter lists on the *training dataset *of 7,000 random protein domain pairs from the PDB40 dataset, which contain both ligand-bound and ligand-free proteins. We validated our results with the SCOP superfamily classification numbers and obtained the ROC and AUC values corresponding to each basic parameter list. The experimental testing and evaluation analysis involved the following steps:

• Perform advanced TOPS+ comparison based on the advanced_SSEArc+Match function for all basic parameters in list *P*_*b*_.

• Compute ROC (Receiver Operating Characteristic) curve analysis for all 7,000 results, and for each parameter list.

• Calculate the AUC values corresponding to the 17 different nC scores.

**Figure 5:**
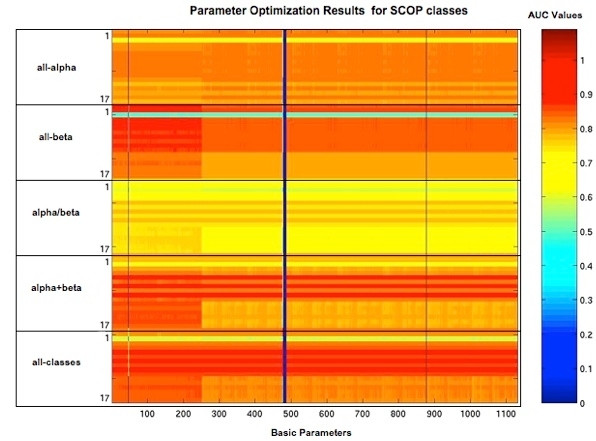
**Parameter optimization results for SCOP classes**. Parameter optimization experimental results for SCOP classes *all-alpha*, *all-beta*, *alpha/beta*, *alpha+beta*, and all classes together. Each element in the matrix represents the AUC values based on the 17 nC scores corresponding to the basic parameters. The *x*-axis represents 1,134 basic parameters and the *y*-axis represents the 17 nC scores from 1 through 17 represented in a block which is repeated five times along the *y*-axis and each block corresponding to the SCOP classes *all-alpha*, *all-beta*, *alpha/beta*, *alpha+beta*, and all classes together.

We have obtained the 17 × 1, 134 AUC values and we plot the results according to the SCOP classes 1-4, which correspond to all-alpha, all-beta, alpha/beta and alpha+beta, and all classes together. In Figure 5, the *x*-axis represents the 1,134 parameters and the *y*-axis denotes the AUC values corresponding to 17 nC scores and for each of the SCOP classes *all-alpha*, *all-beta*, *alpha/beta*, *alpha+beta*, and *all classes *(17 × 5 = 85 points). The color codes adjacent to the actual graph indicate the range of AUC values between 0 and 1. This corresponds to the percentage of accuracy where 0% is represented by blue and 100% by maroon. The first 250 basic parameter lists give the best performance for all the SCOP classes, and the AUC values based on nCE again give consistently better performance for all the parameter values compared to nC scores based on LCS. Specifically, nCEnA(6), nCEnL(8) and nCESAnL(10) give higher AUC values for all SCOP classes and for all the parameter values; while nCLnL(14) and nCLSAnL(16) give higher performance for the all-alpha class. From the parameter tuning table evaluation analysis we have found that the basic parameter *P*_*b *_[P92:3,1,1,1,1] always provides the best result for our training dataset. So we have selected this basic parameter as a default parameter for our advTOPS+ method and performed further analysis and produced the results for PDB40 and Chew-Kedem datasets.

### Datasets

#### PDB40 subset

We have considered the PDB40 representative subset of 4,261 protein domain pairs (which excludes the domain pairs used in our *training dataset*) corresponding to SCOP 1.61, see Table 6. These proteins contain both ligand-bound and ligand-free protein domains. We have compared our advanced TOPS+ method (based on basic parameter value [P92:3,1,1,1,1]) with our basic TOPS+ and TOPS [3,26] methods. The basic TOPS+ comparison method computes the edit distance between two proteins based on TOPS+ strings elements using a dynamic programming approach.

**Table 6:** Structural homology of protein domains for the PDB40 dataset.

SCOP Class	Hom	%	NonHom	%	Total	%
All alpha	129	69	58	31	187	10
All beta	219	68	102	32	321	18
Alpha/beta	452	48	487	52	939	52
Alpha+beta	167	46	193	54	360	20

Total	967	54	840	46	1807	100

Superfamily homologous (Hom) and non-homologous (NonHom) statistics of the PDB40 dataset.

#### Chew-Kedem dataset

We have considered the Chew and Kedem dataset [4,5] to assess the biological significance of our advanced TOPS+ comparison method. This dataset contains 36 medium size representative proteins of five different families: *globins *(17 entries), *alpha-beta *(6 entries), *tim-barrels *(4 entries), *all-alpha *(2 entries), and *all-beta *(7 entries) proteins. We compared our method against the SSAP structure alignment program [9,34,35] and TOPS [3,26] and validated our results based on computation of the *F*-measure [20,21].

### Evaluation Analysis

#### ROC and AUC Analysis

For the PDB40 dataset we have performed evaluation analysis as given below:

• Obtain the pairwise comparison score from the protein comparison method for a given dataset.

• Assignment of Homolog (TP, true positive) and nonHomolog (FP, false positive) based on the SCOP superfamily numbers for each protein domain (see below) and rank them according to the comparison score.

• Perform the Receiver Operating Characteristics (ROC) curve analysis for equation (2). For all the ROC curves we have computed the AUC (Area Under the ROC Curve) values.

#### Homolog vs non-homolog assignment

We have considered the assignment of homologous or non-homologous information of a protein domain pair, based on the standard SCOP classification numbers at superfamily level as an indication of structural homology. If both protein domains belong to same superfamily then they are homologous, otherwise they are non-homologous.

#### F-measure validation analysis for the Chew-Kedem dataset

We have obtained all-against-all comparison scores from all the comparison methods and based on these scores, for each method, we performed pairwise hierarchical clustering using the OC program [36]. We have evaluated the biological significance of the clusters obtained from different protein structure comparison methods based on *F*-measure calculations [20,21].

#### Run Time Analysis

We performed all the analyses using a RedHat 7.2 linux environment with an Intel Pentium IV 1.6 GHz processor. The methods SSAP, TOPS, TOPS+ and advTOPS+ took 9139 s, 75 s, 21 s and 1805 s (s = seconds) respectively to complete 630 pairwise comparisons.

## Authors' contributions

MV developed the advanced TOPS+ comparison method, performed the calculations and prepared the figures. All authors prepared the manuscript, contributed to the discussion, and have approved the final manuscript.

## References

[B1] Gilbert D Westhead DR Viksna J Thornton J A Computer System to Perform Structure Comparison using TOPS Representations of Protein Structure J Comput Chem 2001 26 23 30 10.1016/S0097-8485(01)00096-1 11765848

[B2] Gilbert D Westhead DR Viksna J Frasconi P, Shamir R Techniques for Comparison, Pattern Matching and Pattern Discovery: From Sequences to Protein Topology Artificial Intelligence and Heuristic Methods in Bioinformatics, Volume 183 of NATO Science Series: Computer & Systems Sciences 2003 IOS Press 128 147

[B3] Viksna J Gilbert D Pattern Matching and Pattern Discovery Algorithms for Protein Topologies Algorithms in BioInformatics, Volume 2149 of Lecture Notes in Comput. Sci 2001 Springer-Verlag 98 111

[B4] Krasnogor N Pelta DA Measuring the Similarity of Protein Structures by Means of the Universal Similarity Metric Bioinformatics 2004 20 7 1015 1021 10.1093/bioinformatics/bth031 14751983

[B5] Chew LP Kedem K Finding the Consensus Shape for a Protein Family Algorithmica 2003 38 115 129 10.1007/s00453-003-1045-2

[B6] Goldsmith-Fischman S Honig B Structural Genomics: Computational Methods for Structure Analysis Protein Sci 2003 12 9 1813 1821 10.1110/ps.0242903 12930981 PMC2323979

[B7] Berman HM Westbrook J Feng Z Gilliland G Bhat TN Weissig H Shindyalov IN Bourne PE The Protein Data Bank Nucleic Acids Res 2000 28 235 242 10.1093/nar/28.1.235 10592235 PMC102472

[B8] Orengo CA Taylor WR A Rapid Method for Protein Structure Alignment J Theor Biol 1990 147 4 517 551 10.1016/S0022-5193(05)80263-2 2074728

[B9] Taylor WR Orengo CA Protein Structure Alignment J Mol Biol 1989 208 1 22 10.1016/0022-2836(89)90084-3 2769748

[B10] Russell RB Barton GJ Multiple Protein Sequence Alignment from Tertiary Structure Comparison: Assignment of Global and Residue Confidence Levels Proteins 1992 14 2 309 323 10.1002/prot.340140216 1409577

[B11] Holm L Sander C Protein Structure Comparison by Alignment of Distance Matrices J Mol Biol 1993 233 123 138 10.1006/jmbi.1993.1489 8377180

[B12] Shindyalov IN Bourne PE Protein Structure Alignment by Incremental Combinatorial Extension (CE) of the Optimal Path Protein Engineering 1998 11 9 739 747 10.1093/protein/11.9.739 9796821

[B13] Madej T Mossing MC Hamiltonians for Protein Tertiary Structure Prediction Based on Three-dimensional Environment Principles J Mol Biol 1993 233 3 480 487 10.1006/jmbi.1993.1525 7692069

[B14] Madej T Gibrat JF Bryant SH Threading a Database of Protein Cores Proteins 1995 23 3 356 369 10.1002/prot.340230309 8710828

[B15] Harrison A Pearl F Sillitoe I Slidel T Mott R Thornton J Orengo C Recognizing the Fold of a Protein Structure Bioinformatics 2003 19 14 1748 1759 10.1093/bioinformatics/btg240 14512345

[B16] Grindley HM Artymiuk PJ Rice DW Willett P Identification of Tertiary Structure Resemblance in Proteins Using a Maximal Common Subgraph Isomorphism Algorithm J Mol Biol 1993 229 3 707 721 10.1006/jmbi.1993.1074 8381875

[B17] Koch I Lengauer T Wanke E An Algorithm for Finding Maximal Common Subtopologies in a Set of Protein Structures J Comput Biol 1996 3 2 289 306 10.1089/cmb.1996.3.289 8811488

[B18] Veeramalai M Gilbert D A Novel Method for Comparing Topological Models of Protein Structures Enhanced with Ligand Information Bioinformatics 2008 24 23 2698 2705 10.1093/bioinformatics/btn518 18842602

[B19] Veeramalai M Ye Y Godzik A TOPS++FATCAT: fast flexible structural alignment using constraints derived from TOPS+ Strings Model BMC Bioinformatics 2008 9 358 10.1186/1471-2105-9-358 18759993 PMC2553092

[B20] Handl J Knowles J Kell DB Computational Cluster Validation in Post-Genomic Data Analysis Bioinformatics 2005 21 15 3201 3212 10.1093/bioinformatics/bti517 15914541

[B21] van Rijsbergen CJ Information Retrieval 1979 2 London: Butterworths

[B22] Krishna SS Grishin NV Structural Drift: A Possible Path to Protein Fold Change Bioinformatics 2005 21 8 1308 1310 10.1093/bioinformatics/bti227 15604105

[B23] Harrison A Pearl F Mott R Thornton J Orengo C Quantifying the Similarities within Fold Space J Mol Biol 2002 323 5 909 926 10.1016/S0022-2836(02)00992-0 12417203

[B24] Veeramalai M A Novel Method for Comparing Topological Models of Protein Structures Enhanced with Ligand Information PhD thesis 2005 University of Glasgow 10.1093/bioinformatics/btn518 18842602

[B25] Michalopoulos I Torrance GM Gilbert D Westhead DR TOPS: An Enhanced Database of Protein Structural Topology Nucleic Acids Res 2003 32 D 251 254 10.1093/nar/gkh060 PMC308794 14681405

[B26] Torrance GM Gilbert D Michalopoulos I Westhead DR Protein Structure Topological Comparison, Discovery and Matching Service Bioinformatics 2005 21 10 2537 2538 10.1093/bioinformatics/bti331 15741246

[B27] Westhead D Slidel T Flores T Thornton J Protein structural topology: automated analysis and diagrammatic representation Protein Science 1999 8 897 904 10211836 10.1110/ps.8.4.897 PMC2144300

[B28] Levenshtein VI Binary Codes Capable of Correcting Deletions, Insertions, and Reversals Soviet Physics Doklady 1966 10 8 707 710

[B29] Valiente G Combinatorial Pattern Matching Algorithms in Computational Biology using Perl and R 2009 Taylor & Francis/CRC Press

[B30] Needleman SB Wunsch CD A General Method applicable to the Search for Similarities in the Amino Acid Sequence of two Proteins J Mol Biol 1970 48 3 443 453 10.1016/0022-2836(70)90057-4 5420325

[B31] Smith TF Waterman MS Identification of Common Molecular Subsequences J Mol Biol 1981 147 195 197 10.1016/0022-2836(81)90087-5 7265238

[B32] Mizuguchi K Blundell TL Analysis of conservation and substitutions of secondary structure elements within protein superfamilies Bioinformatics 2000 16 12 1111 1119 10.1093/bioinformatics/16.12.1111 11159330

[B33] Brazma A Jonassen I Vilo J Ukkonen E Pattern Discovery in Biosequences Proc. 4th Int. Coll. Grammatical Inference, Volume 1433 of Lecture Notes in Comput. Sci 1998 Springer-Verlag 257 270

[B34] Orengo CA Brown NP Taylor WR Fast Structure Alignment for Protein Databank Searching Proteins 1992 14 2 139 167 10.1002/prot.340140203 1409565

[B35] Orengo CA Taylor WR SSAP: Sequential Structure Alignment Program for Protein Structure Comparison Methods Enzymol 1996 266 617 635 full_text 8743709 10.1016/s0076-6879(96)66038-8

[B36] Barton GJ OC--A Cluster Analysis Program 2002 http://www.compbio.dundee.ac.uk/downloads/oc/

